# Patient-Reported Outcomes in Patients with Chronic Kidney Disease and Kidney Transplant—Part 1

**DOI:** 10.3389/fmed.2017.00254

**Published:** 2018-01-15

**Authors:** Evan Tang, Aarushi Bansal, Marta Novak, Istvan Mucsi

**Affiliations:** ^1^Multi-Organ Transplant Program, Division of Nephrology, University Health Network, Toronto, ON, Canada; ^2^Institute of Medical Sciences, University of Toronto, Toronto, ON, Canada; ^3^Centre for Mental Health, University Health Network, Toronto, ON, Canada; ^4^Department of Psychiatry, University of Toronto, Toronto, ON, Canada

**Keywords:** patient-reported outcomes, chronic kidney disease, kidney transplantation, renal transplantation, patient-reported outcome measures, quality of life measurement, quality of life

## Abstract

Chronic kidney disease (CKD) is a complex medical condition that is associated with several comorbidities and requires comprehensive medical management. Given the chronic nature of the condition, its frequent association with psychosocial distress, and its very significant symptom burden, the subjective patient experience is key toward understanding the true impact of CKD on the patients’ life. Patient-reported outcome measures are important tools that can be used to support patient-centered care and patient engagement during the complex management of patients with CKD. The routine collection and use of patient-reported outcomes (PROs) in clinical practice may improve quality of care and outcomes, and may provide useful data to understand the disease from both an individual and a population perspective. Many tools used to measure PROs focus on assessing health-related quality of life, which is significantly impaired among patients with CKD. Health-related quality of life, in addition to being an important outcome itself, is associated with clinical outcomes such as health care use and mortality. In Part 1 of this review, we provide an overview of PROs and implications of their use in the context of CKD. In Part 2, we will review the selection of appropriate measures and the relevant domains of interest for patients with CKD.

## Introduction

In developed countries, there has been a rapid increase in the prevalence of chronic non-communicable diseases, which are responsible for the majority of morbidity and premature deaths in these societies (1, 2). These health conditions require complex long-term professional care and self-management (1, 2). Within the context of the health care system, management of chronic health conditions has been shifting away from the traditional provider-directed care model to a patient-centered care model (3–5). Patient-centered care utilizes the perspective of patients and allows them to play a key role in defining and managing their care (3–5). It promises a more holistic approach, improved patient experience, as well as improved medical outcomes compared with traditional care models (3, 5–12). Incorporating the patient perspective into clinical practice could improve the accuracy and completeness of assessment, provides accountability, and may play a role in outcome prognostication (13–15).

Chronic kidney disease (CKD) represents a major public health problem. Its prevalence has been rapidly increasing, in part, due to the rising prevalence of obesity, diabetes and hypertension (16–21). CKD is associated with substantial disease burden as both the disease and its various treatments—dietary and lifestyle modifications, renal replacement therapies (dialysis and kidney transplant)—are very intrusive. Accordingly, CKD is associated with substantially impaired quality of life (QOL) and a significant increase in the risk of cardiovascular disease and premature death (22–25).

Patient-reported outcome measures (PROMs) are direct responses from patients without alteration or interpretation by a clinician (12, 14). Use of PROMs can improve the assessment of disease burden among patients with CKD (12, 14). As such, incorporation of PROMs in routine clinical care would be an important tool toward facilitating patient-centered care (26–29). Moreover, involving patients in defining clinical and research priorities has the potential to enhance the relevance and acceptability of research from the perspective of all stakeholders, including patients, the public, clinicians, funding and regulating agencies, and policy makers (29, 30) In addition, establishing a shared platform of understanding may improve patient-provider communication, increase adherence, and improve clinical outcomes (31, 32). Perhaps the most crucial component of patient engagement is incorporating the lived patient experience in health care delivery.

There has been an increasing interest in the systematic collection of patient-reported outcomes (PROs) data for monitoring the impact of chronic illness and improving care among patients with chronic medical conditions such as cancer, rheumatoid arthritis, chronic obstructive pulmonary disease, and heart failure (14, 15, 33–35). Patient-reported measures (PRMs) have been shown to be superior to clinician reports both in the detection of symptoms and side effects of treatment, while also, being more sensitive to changes in functional status compared with standard of care (14, 36–39).

Using electronic data capture to assess PROMs may improve the feasibility of assessing PROMs in routine clinical practice (40–42). It eliminates the need for subsequent data entry, storage of the questionnaires, and reduces the risk of privacy breach. It has the potential for immediate scoring and presentation of the results (43–45), offers the potential to link PROMs with clinical data in electronic health records (46, 47), to enhance communication in multidisciplinary care (48, 49), and to facilitate the assessment of PROMs independently from patient-provider encounters (43, 44, 48, 50). Electronic collection of PROs has been linked to improved QOL, reduced rates of hospitalization, and increased adherence and survival among a large cohort of outpatients receiving chemotherapy for advanced cancer (39). These findings support the use and potential implications of using PROs in CKD management (39, 47).

The aim of this review is to explore the use of PROs in the CKD population. In Part 1, we will describe PROs and implications of use. In Part 2, we will review the selection of appropriate PROMs and the relevant domains of interest for patients with CKD.

## Chronic Kidney Disease

### Defining CKD

Chronic kidney disease is a chronic, frequently progressive condition, defined by structural or functional abnormalities of the kidney and/or a reduced glomerular filtration rate (GFR) of less than 60 mL/min/1.73 m^2^ for more than 3 months (51–55). It is caused by many different conditions including diabetes mellitus, hypertension, glomerulonephritis, genetic diseases, drug toxicity, urological conditions, infections, and acute kidney injury, among others (55, 56). CKD is a heterogeneous condition due to the varying severity and risk of progression, cause and pathology of disease, and comorbidities experienced by individual patients (57).

The classification of CKD is based on GFR (G category) and abnormal urinary albumin excretion (A category) (10, 54, 55, 58, 59). CKD is divided into five GFR stages, ranging from G1, representing normal to high kidney function with evidence of kidney disease, to G5 (GFR < 15 mL/min/1.73 m^2^), known as end-stage kidney disease (ESKD). Patients with various stages of CKD can be managed by lifestyle changes and medications with the aim of slowing disease progression (53, 55, 59). ESKD, however, is potentially life threatening without receiving renal replacement therapy (RRT) in the form of dialysis (peritoneal or hemodialysis) or kidney transplantation (10, 52, 60). Classification guidelines recommend the use of a suffix to identify the RRT modality in patients with stage 5 CKD; with “D” representing dialysis and “T” representing a functioning kidney transplant (52, 55). The stages of CKD enable classification of the severity of renal impairment with existing guidelines in place aimed at management of each stage (10, 54, 55).

### Epidemiology and Significance of CKD

The latest estimates indicate that the prevalence of CKD is about 14% in Americans, 12.5% in Canadians, and 8–16% globally (61–64). CKD continues to be a growing public health concern for several reasons. Firstly, CKD is frequently caused by diabetes, hypertension, and obesity, with the prevalence of these conditions increasing (16–20, 55, 56, 65). As a result, the incidence of CKD is expected to rise simultaneously (19, 66, 67). Progressive aging of the CKD population is associated with increasing morbidity burden (19, 54, 58, 65, 67, 68), resulting in high rates of mortality (23–25, 69, 70) and severely impaired QOL (22, 71, 72). Often underdiagnosed and undertreated, the psychosocial distress associated with CKD further increase the morbidity and disease burden in this patient population (73–77). Globally, these trends pose dramatic consequences for health care financing and delivery systems (78).

In addition to the human suffering, management of CKD is costly (79, 80). In the United States, the annual cost of each patient undergoing dialysis ranges between 26,000 and 85,000 US dollars, depending on type of dialysis (60, 62, 79, 81). Comparatively, the average annual cost of dialysis per patient in Canada is estimated to be approximately 60,000 Canadian dollars (or approximately 45,000 US dollars) (82). As the prevalence of CKD increases, the associated cost burden will also continue to rise (78, 79).

Kidney transplantation has emerged in the past decade as the gold standard of RRT as it is associated with improved QOL (70, 83–85), decreased morbidity (70, 84), and decreased mortality (20, 70, 86, 87). Furthermore, transplantation is also substantially more cost-effective than maintenance dialysis (78, 79, 88–90). However, a global shortage of available organs prevents kidney transplant from being a universally accessible treatment modality (67, 91).

### CKD as a Chronic Condition

Although patients with CKD can be managed with medications and RRT, these treatments will not reinstate normal kidney function. The primary goals of medical treatment during earlier stages of the disease are to prevent or slow disease progression, reduce the consequences of CKD, and to detect and manage subsequent complications (51, 57, 60). Complications and consequences include anemia, fatigue, sleep disorders, mental health conditions (such as depression and anxiety), bone and mineral disorders, and cardiovascular diseases (22, 76, 92, 93). Ultimately, as patients proceed through the stages of CKD, there is a marked increase in symptom burden, impairment of QOL (22, 94), and an increase in morbidity (22, 71, 95). Consequently, management of CKD presents a unique challenge as the needs of patients change as the disease progresses through its various stages (96–98).

## Patient-Reported Measures

Traditional clinical tests do not adequately assess the health status, well-being, and functional capacity of patients as patients with similar clinical severity may have vastly different responses to the disease process (99, 100). Likewise, biomedical characteristics and biomarkers are often inadequate for predicting the subsequent illness trajectory (101, 102) or response to a specific treatment. Incorporating the patient experience and patient perspective can improve the accuracy and completeness of assessment and may improve the prediction of outcomes (13, 40–42, 103, 104). These observations led to the rise of health frameworks with patient-centered care as the focus, offering a much more holistic approach to the measurement of outcomes (3, 5–12).

### Quality of Life

The first studies focusing on patient-centered care measured QOL: the Almeda County study (105), the RAND Health Insurance study (106), and the Medical Outcomes study (107, 108). These studies represented a major shift in ideology toward one, which took patient’s priorities into consideration. More recently, the World Health Organization defines QOL as “the individual’s perception of their position in life in the context of the culture and value systems in which they live and in relation to their goals, expectations, standards, and concerns (109). Recognizing the breadth of this definition, the medical community further refined this concept in the context of health and coined the term health-related quality of life (HRQOL) (108).

### Health-Related Quality of Life

There are various definitions of HRQOL in the literature and their detailed comparative assessment is beyond the scope of this review. For the purpose of this review, HRQOL is defined as the “impact of disease and treatment across the physical, psychological, social and somatic domains of functioning and well-being” (108, 110). Measuring HRQOL offers a patient’s subjective perspective on their understanding and experience of the disease and/or its treatment on their overall health and well-being (111, 112). Critics of HRQOL claim that there is a lack of conceptual clarity and measurement feasibility (113). Recent advancement in patient-centered care has striven to address the aforementioned issues and have been broadly classified as PRMs (12, 14).

### Patient-Reported Measures

Although QOL, HRQOL, PROMs, and PRMs are often used interchangeably, these terms have important distinctions regarding dimensionality and scope (34, 108). A PRM is any measure that is reported directly by patients without interpretation from a clinician or other health care provider (12, 14). PRMs carry a broader scope in comparison to HRQOL by collecting any information reported by the patient, beyond QOL or HRQOL alone (13, 114, 115).

Patient-reported measures can be classified into two different categories: PROs, referring to one’s perception of health status, and patient-reported experiences (PREs), referring to one’s perception of the care they received (12, 14, 35, 116, 117). Tools designed to measure PROs and PREs are thus labeled as PROMs or patient-reported experience measures (PREMs) (12, 14, 116, 117).

Patient-reported outcome measures can be used to assess a wide variety of health-relevant concepts and are indispensable for gathering comprehensive information about a patient (12, 14). There are four overarching categories of information that PROMs can assess: HRQOL, functional status, symptoms and symptom burden, and health behaviors and perceptions (13, 14). In contrast, PREMs provide an evaluation of quality of health care delivery from the patient’s perspective, such as facility cleanliness, access to information and health care teams, communication, support received, and transportation (12). A core tenant of PROMs and PREMs requires that responses are received without any interpretation as this enables an accurate and unadulterated account about the patient.

### Utility of PRMs in Clinical Practice

While the use of PROMs in clinical practice is gaining increasing popularity, the utility of PREMs has been the subject of debate (12, 14, 35, 118, 119). This debate was driven by the inclusion of PREMs as a metric for governmental health care reimbursement in the United States, which resulted in private insurers following suit (12, 119, 120). Subsequently, several studies have suggested that PREMs have limited utility as a marker of quality of care, with PREMs being shown to have little to no association with quality of care or clinical outcomes (35, 118, 120, 121). PROMs, on the other hand, have demonstrated significant association with clinical outcomes (12, 14, 103, 104, 122).

Measuring PROMs during clinical encounters provides an opportunity for patients to reveal physical, psychological, or social concerns that might have an impact on their daily life. These might be concerns not discussed, overlooked, or underestimated by the health care team (36, 112). They also enable the medical team to accurately assess and quantify symptom burden and HRQOL: vital components of the chronic illness experience. Integration of PROMs within the clinical care of patients with CKD has the potential to improve the lives of individual patients and also to understand care needs on the population level (40, 41, 123). Recognition of the importance of PROMs have resulted in the development of guidelines by national organizations to provide guidance on the implementation and reporting of PROMs (117, 124). Since PROMs have shown increased utility and implications for clinical research, the remainder of this paper will focus on PROMs.

## Patient-Reported Outcome Measures

### Structure of PROMs

Patient-reported outcome measures are composed of individual questions or statements (with standardized response options), also known as items. Items are related to particular domains, which are overarching, measurable themes of interest (e.g., physical functioning, emotional well-being, etc.). Certain PROMs have algorithms in place to aggregate individual items that are similar, into specific composite ratings or domain scores. For instance, one PROM frequently used in CKD is the Kidney Disease Quality of Life Short Form (KDQOL-SF) (88, 125, 126). The general domains include the following: physical functioning, physical role functioning, bodily pain, general health perceptions, vitality, emotional role functioning, social role functioning, and mental health and additional, kidney disease-related domains, such as effects of kidney disease, burden of kidney disease, symptom list, sleep problems—each with scores ranging from 0 to 100 (125, 126). In addition, physical and mental health composite scores are generated, also ranging from 0 to 100, with higher scores representing better overall physical or mental health, respectively (126).

### PROMs in CKD

Various PROMs have been developed and validated in the CKD population and can typically be classified as a generic instrument or a CKD-specific instrument (92, 127–130). Generic instruments are PROMs that measure various aspects of patients’ health status and can be administered across the general population or in any patient populations (see Table 1). This is advantageous as it allows for different populations to be more readily compared; however, the variance in burden of disease in certain populations can result in a ceiling or floor effect (127, 131–133). Ceiling or floor effects occur when subjects score at the bottom (floor effect) or at the top (ceiling effect) of the range of a scale, usually because the items are too easy or too difficult. This means that the scale cannot discriminate individuals even with different amounts of the measured trait above or below those points. In addition, because generic tools do not assess disease specific characteristics, they are less sensitive to the severity of disease and may be less responsive to change in the condition in response to treatments or as the condition progresses (134). Some commonly known generic instruments include the Medical Outcomes Study Short Form (SF-36, SF-20, SF-12) (129, 135–139), Quality of Well Being Scale (140), Sickness Impact Profile (SIP) (141, 142), EuroQol (143, 144), Patient-Reported Outcomes Measurement Information System (PROMIS-57, PROMIS-43, PROMIS-29) (33, 115), World Health Organization Quality of Life Scale (WHOQOL-BREF) (145, 146), Illness Intrusiveness Rating Scale (IIRS) (147–149), Patient Health Questionnaire (PHQ) (150, 151), Social Difficulties Inventory (SDI) (152), Generalized Anxiety Disorder 7-item Scale (GAD-7) (153), Edmonton Symptom Assessment System (ESAS) (154–156), Epworth Sleepiness Scale (ESS) (157), Cambridge-Hopkins diagnostic questionnaire for Restless Leg Syndrome (CH-RLSq) (158), and Health Utility Index (88, 127, 129, 133, 135, 136, 159, 160).

**Table 1:** Selected generic PROMs and their measured domains.

	Physical					Mental			Social			
	Pain	Physical function	Usual activities	Fatigue	Other physical	Anxiety	Depression	Other mental health	Social functioning	Role function	Other social	General health
EQ5D (144)	Pain or discomfort	Mobility	Usual activities Self-care			Anxiety	Depression					General health

HUI2 (88)	Pain	Mobility	Self-care		Sensation Fertility			Emotion Cognition				

IIRS (147–149, 161)					Physical well-being and diet						Work and finance marital, sexual, family relationships recreation and social relations	Other aspects of life

SIP (141, 142)		Physical composite						Psychosocial composite				General health

WHOQOL-BREF (145, 146)		Physical health						Psychological			Social relationships Environment	

QWB (140)		Physical functioning Mobility	Performance of usual activities Self-care									Acute and chronic symptoms and problems

ESASr (154–156)	Pain			Tiredness	Drowsiness Nausea Appetite Shortness of breath	Anxiety	Depression					Well-being

SF-36 (129, 135–139)	Bodily pain	Physical functioning			Vitality			Mental health	Social functioning	Role limitations due to emotional or physical problems		General health perception

PROMIS-57 (43 & 29) (33, 115)	Pain Pain interference	Physical function		Fatigue	Sleep disturbance	Anxiety	Depression			Ability to participate in social roles		

PHQ (150, 151)							Depression					

GAD-7 (153)						Anxiety						

CH-RLSq (158)					Restless leg syndrome							

ESS (157)					Sleep propensity							

ISI (162)					Insomnia							

FACIT-fatigue (163)				Fatigue								

SDI (152)			Everyday living						Social distress		Self and others money matters	

*EQ5D, European Quality of Life 5 Dimensions; HUI, Health Utility Index; IIRS, Illness Intrusiveness Rating Scale; SIP, Sickness Impact Profile; WHOQOL-BREF, World Health Organization Quality of Life; QWB, Quality of Well Being Scale; ESASr, Edmonton Symptom Assessment System revised; SF-36, Medical Outcome Study 36-Item Short-Form Survey; PROMIS-57 (43 & 29), Patient-Reported Outcomes Measurement Information System 57/43/29 Question Short Form; PHQ, Patient Health Questionnaire; GAD-7, Generalized Anxiety Disorder 7-Item scale; CH-RLSq, Cambridge-Hopkins diagnostic questionnaire for Restless Leg Syndrome; ESS, Epworth Sleepiness Scale; ISI, Insomnia Severity Index; FACIT-Fatigue, Functional Assessment in Cancer Intervention and Therapy; SDI, Social Difficulties Inventory*.

Chronic-kidney-disease-specific instruments are PROMs that are tailored to the specific symptom burden and disease experience by patients with CKD (see Table 2) (22, 116, 127, 159). These instruments are often very specific, thus avoiding the ceiling or floor effects observed when using generic instruments (127, 132). The specificity of the questions, however, usually precludes its use in other populations thus limiting generalizability of results (127, 133). Some commonly known CKD-specific instruments include the following: Kidney Disease Quality of Life (KDQOL) (136, 164), Quality of Life Index Dialysis version (QLI-D) (165, 166), Kidney Disease Questionnaire (KDQ) (167), Kidney Transplant Questionnaire (KTQ) (168), Renal Quality of Life Profile (RQLP) (169), CHOICE Health Experience Questionnaire (CHEQ) (170), End-Stage Renal Disease Symptom Checklist–Transplant Module (ESRD-SCL-TM) (171), and Renal Dependent Individualized Quality of Life Questionnaire (125, 127, 136, 170, 172–174). By no means exhaustive, a more comprehensive list of PROMs will be explored in Part 2 of our review. Despite the wide selection of available PROMs, we are not aware of routine PROM use in nephrology clinical settings currently.

**Table 2:** Selected CKD-specific PROMs and their measured domains.

	Physical			Mental				Psychosocial				
	physical function	Symptoms	Disease impact	Other physical	Emotion	Cognition	Mental health	Social functioning	Role function	Other psychosocial	Other social	General health
KDQOL-36 (136, 164)	Physical composite	Symptoms and problems	Effects and burden of kidney disease				Mental composite					

KDQOL-SF (136, 164)	Physical composite	Symptoms and problems, Sexual function, Sleep	Effects and burden of kidney disease	Energy/fatigue, Pain	Emotional well-being	Cognitive function	Mental composite	Quality of social interaction Social functioning	Role limitations due to emotional problems or physical problems		Work status, Social supportDialysis staff encouragementPatient Satisfaction	General health perceptions

QLI-D (165, 166)	Health and functioning									Psychosocial/spiritual	Social and economic family	Quality of life

RQLP (169)	Physical activity		Impact of treatment Eating and drinking							Psychosocial activities	Leisure time	

CHEQ (170)	Physical functioning	Sexual functioning sleep	Diet	Vitality, Body image, Bodily pain		Cognitive functioning	Mental health	Social functioning	Role limitations due to emotional problems or physical problems		Work, Recreation, Travel FinancesDialysis access, Freedom	General quality of life

RDI-QLQ (174)	Physical functioning	Sex life	Enjoyment of food Restriction of fluid intake	Physical appearance					Family life	Worries about the future motivation to achieve things spiritual/religious	Social life and friendships work holidays and leisure Activities dependency freedom social prejudice	

KDQ (167)		Physical symptoms		Fatigue	Frustration		Depression				Relationships	

KTQ (168)		Physical symptoms		Fatigue	Emotions Uncertainty Fear							

ESRD-SCL-TM (171)	Physical capacity	Cardiac and renal impairment	Side effect of corticosteroids	Increased hair and gum growth		Cognitive capacity	Transplant associated psychological distress					

*KDQOL-36/KDQOL-SF, Kidney Disease Quality of Life 36-Item Question/Short Form; QLI-D, Quality of Life Index Dialysis version; RQLP, Renal Quality of Life Profile; CHEQ, CHOICE Health Experience Questionnaire; RDI-QLQ, Renal Dependent Individualized Quality of Life Questionnaire; KDQ, Kidney Disease Questionnaire; KTQ, Kidney Transplant Questionnaire; ESRD-SCL-TM, End-Stage Renal Disease—Symptom Checklist—Transplant Module*.

## Implications of PROMs in Clinical Care

Measurement of PROs provides information about patient health and well-being which cannot be obtained by traditional medical/biological assessments (11, 12, 14). Responses through a subjective lens, without clinician interpretation, facilitate a holistic approach by gaining insight into patients’ values and preferences while also obtaining reports on outcomes of care (11, 12, 31, 32). An important goal of the extensive research in PROMs would be the eventual integration of tools into the standard of care of patients with CKD. There are many implications for clinical practice based on the results drawn from PROMs. Integration of PROMs can enhance patient-centered care, facilitate communication between patients and health care providers as well as between health care team members, and provide information for evaluating clinical outcomes (3, 6, 9, 40, 41, 119).

### Enhanced Patient-Centered Care

Patient-reported outcome measures can be used to guide treatment options by elucidating domains that require intervention, especially those that are frequently under-assessed or neglected by the managing health care teams (9, 175). For instance, the identification of psychological distress, sleep problems, fatigue, social difficulties, and/or pain interference scores or low physical function scores can assist the health care team in tailoring treatment to target these areas (7, 11). Beyond merely identifying domains, PROMs can assist to determine the level of severity within each domain. Periodic collection of PROMs allows the health care team to track the progression of symptoms and subjective health status, as well the impact of different treatment modalities (176). Longitudinal assessment of PROMs can further be utilized in a “treat to target” approach, where treatment and referrals are prescribed with the aim of achieving a specific score (177, 178). Major deviations from baseline scores can also serve as an indicator for interventions.

Patient-reported outcome measures can also be utilized to identify which symptoms are personally relevant and important to patients, which is an essential component of assessing the impact of disease and their QOL (179). Considering the recent finding that 80% of current clinical research does not include the top 10 research priorities as identified by patients on or nearing dialysis, understanding patients’ needs, values, and opinions are crucial to improving outcomes (9, 27, 31, 32). As such, PROMs can be used to address this disparity in current research, by utilizing information from PROMs to align research priorities with patient priorities (26, 31–33).

### Enhanced Communication

The use of PROMs can facilitate better communication between the patient and provider. This is possibly due to PROMs providing an avenue to broach on topics related to specific domains, such as adverse events, anxiety, or depression, which may otherwise not be communicated openly by patients or overlooked by the health care team (83, 180–182). Other findings suggest that use of PROMs improves patient engagement by engaging patients in treatment planning and evaluation (182, 183). In addition, Schorn et al. demonstrated that use of PROM in primary care improved self-management behavior (183). Ultimately, this has the potential to improve patient outcomes as seen in the study conducted by Basch et al., where patients randomized to the group using PROMs expressed a greater number of symptoms and had better overall survival and less hospitalization compared with the group receiving standard care (39).

### Clinical Outcomes

Paramount to the discussion of implication is that of “hard” clinical outcomes, such as mortality, hospitalization, and symptom burden. Many studies have suggested that PROs have the potential to be used for prognostication (103, 104, 184–187). While links between clinical outcomes and PROs have been identified, only a few studies have explored the impact of interventions on these outcomes (188). Further studies are required to elucidate the impact of PRO modification on clinical outcomes.

## Conclusion

In this paper, we have reviewed the relevance and importance of using PROMs in the context of CKD, largely supported by the need to address currently unexplored aspects of the illness experience of patients suffering from the multifaceted and chronic nature of this disease, which contribute to a high symptom burden and clinical outcomes. This includes both physical and psychosocial concerns, which are often overlooked in this patient population. As such, the value of patient involvement in care delivery and research is increasingly recognized. PROMs are the latest frontier as a means of collecting and utilizing patient-reported information without clinician interpretation. This not only provides a means to identify and address the patient concerns but also has benefits including the facilitation of patient-centered care, enhancing communication, and elucidating research priorities. PROMs, however, are still a growing field with implications for clinical practice that is still being uncovered in the context of CKD. In Part 2, we will explore the domains of interest for patients with CKD and the selection of appropriate PROMs.

## Author Contributions

ET and AB were involved in the conception of the review, including a critical review of the literature accompanied by the drafting of the manuscript. MN and IM provided support in the form of professional appraisal and the provision of supporting literature. Critical revisions were performed and the final version was reviewed and approved by both IM and MN.

## Conflict of Interest Statement

The authors declare that this review was conducted without any commercial or financial conflict of interest.
